# Is One Anastomosis Gastric Bypass with a Biliopancreatic Limb of 150 cm Effective in the Treatment of People with Severe Obesity with BMI > 50?

**DOI:** 10.1007/s11695-021-05499-3

**Published:** 2021-06-26

**Authors:** Arnaud Liagre, Francesco Martini, Radwan Kassir, Gildas Juglard, Celine Hamid, Hubert Boudrie, Olivier Van Haverbeke, Laura Antolino, Tarek Debs, Niccolo Petrucciani

**Affiliations:** 1 Ramsay Générale de Santé, Clinique des Cedres, Bariatric Surgery Unit, Cornebarrieu, France; 2 Department of Digestive Surgery, CHU Félix Guyon, Saint Denis, La Réunion France; 3 Department of Medical and Surgical Sciences and Translational Medicine, Faculty of Medicine and Psychology, St. Andrea Hospital, Sapienza University, Rome, Italy; 4 Department of Digestive Surgery and Liver Transplantation, Nice University Hospital, Nice, France

**Keywords:** Bariatric surgery, One anastomosis gastric bypass, Severe obesity, BMI > 50

## Abstract

**Purpose:**

The treatment of people with severe obesity and BMI > 50 kg/m^2^ is challenging. The present study aims to evaluate the short and mid-term outcomes of one anastomosis gastric bypass (OAGB) with a biliopancreatic limb of 150 cm as a primary bariatric procedure to treat those people in a referral center for bariatric surgery.

**Material and Methods:**

Data of patients who underwent OAGB for severe obesity with BMI > 50 kg/m^2^ between 2010 and 2017 were collected prospectively and analyzed retrospectively. Follow-up comprised clinical and biochemical assessment at 1, 3, 6, 12, 18, and 24 months postoperatively, and once a year thereafter.

**Results:**

Overall, 245 patients underwent OAGB. Postoperative mortality was null, and early morbidity was observed in 14 (5.7%) patients. At 24 months, the percentage total weight loss (%TWL) was 43.2 ± 9, and percentage excess weight loss (%EWL) was 80 ± 15.7 (184 patients). At 60 months, %TWL was 41.9 ± 10.2, and %EWL was 78.1 ± 18.3 (79 patients). Conversion to Roux-en-Y gastric bypass was needed in three (1.2%) patients for reflux resistant to medical treatment. Six patients (2.4%) had reoperation for an internal hernia during follow-up. Anastomotic ulcers occurred in three (1.2%) patients. Only two patients (0.8%) underwent a second bariatric surgery for insufficient weight loss.

**Conclusion:**

OAGB with a biliopancreatic limb of 150 cm is feasible and associated with sustained weight loss in the treatment of severe obesity with BMI > 50 kg/m^2^. Further randomized studies are needed to compare OAGB with other bariatric procedures in this setting.

**Graphical abstract:**

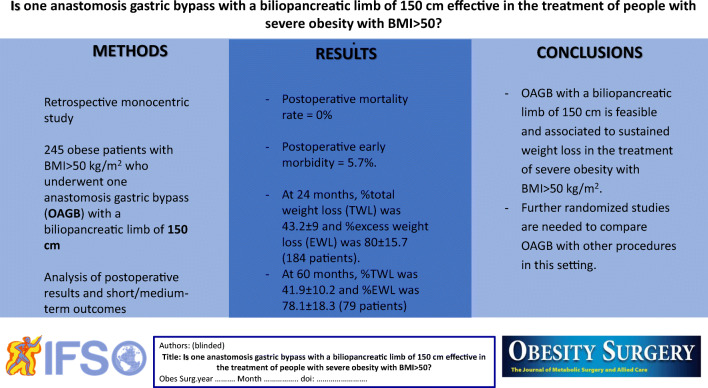

## Introduction

Obesity is a worldwide epidemic, and bariatric surgery has proven to be the most effective treatment for severe obesity [1]. Some authors have used the word super-obesity to define as a body mass index (BMI) > 50 kg/m^2^ and super super-obesity to define a BMI > 60 kg/m^2^ [2]. Recent studies associate bariatric surgery with a considerable long-term insufficient weight loss or complications, with a secondary procedure needed in approximately 20% of patients [3, 4]. The initial BMI is a significant predictive factor of insufficient weight loss, with worse weight loss results in patients with a higher initial BMI [5, 6]. The treatment of people with severe obesity and BMI > 50 kg/m^2^ is challenging for three main reasons: (1) insufficient weight loss is more frequent after bariatric surgery in this setting; (2) surgery is technically more demanding; and (3) surgery is associated with higher postoperative morbi-mortality [7].

Several bariatric procedures have been proposed to treat severe obesity with BMI > 50 kg/m^2^, including Roux-en-Y gastric bypass (RYGB), one anastomosis gastric bypass (OAGB), single anastomosis duodeno-ileal bypass (SADI), sleeve gastrectomy (SG), and biliopancreatic diversion with duodenal switch (BPD-DS) [8–15]. There is still no robust evidence supporting the choice of one procedure over the others.

OAGB was recognized as a mainstream bariatric procedure by the International Federation for the Surgery of Obesity and Metabolic Disorders (IFSO) in 2018 [16]. Since then, the efficacy and safety of OAGB in treating obesity and its related associated medical problems have been demonstrated in several studies that have included thousands of patients [17–20]. A randomized study that compared OAGB with RYGB demonstrated the non-inferiority of OAGB in weight loss and metabolic improvement at 2 years [21]. OAGB with a biliopancreatic limb of 150 cm has produced similar outcomes with a better safety profile to those seen with a limb of 200 cm [22–24].

The present study aims to evaluate the short and mid-term outcomes of OAGB with a biliopancreatic limb of 150 cm as a primary bariatric procedure to treat people with severe obesity and BMI > 50 kg/m^2^ in a referral center for bariatric surgery.

## Materials and Methods

### Patient Selection

The Institutional Review Board of our institution approved the study, which is registered as IORG-IRB: IORG0009085 COS-RGDS-2019-11-001-LIAGRE-A. All people with a BMI > 50 kg/m^2^ who underwent OAGB with a biliopancreatic limb of 150 cm as a primary bariatric procedure between May 2010 and December 2017 were identified retrospectively from a prospective database that included all patients who underwent bariatric surgery in our department. Data from our database, computerized hospital records, and case notes were obtained when necessary. Data were further supplemented by contacting the patients and their general practitioners if needed.

### Preoperative Workup

Indications for primary surgery were in line with the National Health Authority (Haute Autorité de Santé, HAS) recommendations, and surgery was proposed as a second-line treatment after 6–12 months of medical management [25]. Preoperative workup included upper gastrointestinal (GI) endoscopy, abdominal ultrasound, clinical, biochemical, nutritional, and psychological assessment. The multidisciplinary obesity board of the institution validated the indication for surgery.

### Surgical Technique

All the patients underwent OAGB with a biliopancreatic limb of 150 cm. The stomach was sectioned at the level of the incisura angularis and calibrated on a 36 Fr bougie to fashion a long and narrow pouch. An antecolic laterolateral gastrojejunal anastomosis was created using a stapler with a 60-mm vascular cartridge. No closure of mesenteric defects was performed.

### Postoperative Outcomes and Follow-Up

Postoperatively, water intake was started the evening of surgery, and a semi-liquid diet was allowed on postoperative day 1. Postoperative complications were classified according to the Clavien–Dindo classification [26]. Proton pump inhibitors (PPIs) were prescribed for 3 months after surgery. After this period, the PPIs were continued only in response to gastroesophageal reflux disease (GERD) symptoms. Micronutrient supplementation was administered routinely to all patients, as previously reported [24]. After 2018, we administered a poly-vitamins capsule designed to avoid nutritional issues after OAGB. We choose this formula as it consists in a single product, which may improve patients’ compliance.

Weight loss outcomes were expressed as percentage total weight loss (%TWL) and percentage excess weight loss (%EWL), and calculated as [initial weight − follow-up weight] × 100 and [initial weight − follow-up weight] × 100/[initial weight − ideal weight], respectively. Ideal weight was set as that equivalent to a BMI of 25 kg/m^2^. Follow-up continued with clinical and biochemical assessment at 1, 3, 6, 12, 18, and 24 months postoperatively and once a year thereafter.

The evolution of obesity-related associated medical problems was assessed according to the use and discontinuation of medication postoperatively in the instance of diabetes, hypertension, dyslipidemia, and osteoarthritis. Remission of hypertension was defined as a systolic blood pressure of less than 130 mmHg or diastolic blood pressure of less than 85 mmHg without the use of antihypertensive drugs. Improvement was defined as a decrease in the quantity or dosage of antihypertensive drugs. Diabetes remission was defined as fasting glucose of less than 5.6 mmol/L and a glycosylated hemoglobin value of less than 6.5% without the use of oral hypoglycemic medications or insulin. Improvement was defined as a decrease in the quantity or dosage of oral hypoglycemic medications or insulin. Improvement of osteoarthritis was evaluated based on symptoms, mobility, and use of painkillers. The presence of preoperative sleep apnea syndrome was quantified by sleep studies and postoperative resolution by discontinued use of continuous positive airway pressure masks. Gastrointestinal and endocrinological complications included diarrhea, hypoglycemia, abdominal pain, and GERD. Biliary reflux was defined as the presence of clinical symptoms necessitating treatment, such as heartburn and/or bile vomiting and/or biliary regurgitation, particularly during the night or in dorsal decubitus.

### Data Presentation and Statistical Analysis

Continuous data are reported as means, standard deviations, and ranges. Nominal data are expressed as numbers and percentages. Comparisons were made using the χ^2^ test for nominal data or Student’s *t* test for continuous data. The paired Student’s *t* test was used to compare preoperative and postoperative biochemical values. A P value of ≤ 0.05 was considered to be statistically significant. All statistical analyses were performed using SPSS software version 25.

## Results

### Patients Characteristics and Surgical Procedures

During the study period, 245 patients with BMI > 50 kg/m^2^ underwent OAGB as a primary procedure. The characteristics and associated medical problems of patients are listed in Tables 1 and 2, respectively. Surgical procedures associated with OAGB were hiatal hernia repair with cruroplasty in eight (3.2%) patients. Major steatosis was detected in 40 (16.3%) cases.

**Table 1:** Characteristics of the included patients before surgery and at follow-up

Variable	Baseline	12 months	24 months	60 months	72 months	80 months
N.	245	215	184	79	43	14
Female sex	170 (79%)					
Age (years)	39.7 ± 13.2 (18–71)					
Lost to F-U		6/215 (3%)	8/184 (4%)	11/79 (14%)	6/43 (14%)	3/14 (21%)
Weight (kg)	150 ± 22.3	94 ± 18.5	85 ± 18.2	86 ± 17.8	88.6 ± 18.2	87.9 ± 21
	(107–250)	(56–170)	(53–165)	(60–140)	(60–132)	(65–135)
BMI (kg/m^2^)	54 ± 4.9	34.5 ± 6.1	31.1 ± 5.3	31.6 ± 5.6	32.1 ± 6.3	30.5 ± 6.5
	(50–75)	(24–74)	(22–50)	(23–48)	(23–49)	(23–41)
%EWL		69.4 ± 16	80 ± 15.7	78.1 ± 18.3	76 ± 19.9	80.5 ± 21
		(18–103)	(17–112)	(30–106)	(26–106)	(50–106)
%TWL		37.7 ± 8.4	43.2 ± 9	41.9 ± 10.2	41.5 ± 11.5	42.9 ± 11.8
		(11–58.4)	(5–65)	(17.6–64)	(14–64)	(27–64.6)
Class I obesity		80/215	66/184	25/79	11/43	1/14
		(37.2%)	(35.8%)	(31.6%)	(25.5%)	(7.1%)
Class II obesity		62/215	21/184	5/79	5/43	2/14
		(28.8%)	(11.4%)	(6.3%)	(11.6%)	(14.2%)
Class III obesity		32/215	16/184	20/79	6/43	2/14
		(14.9%)	(8.6%)	(12.6%)	(13.9%)	(14.2%)
Treated	97/245	25/215		15/78		
GERD	(39.5%)	(25%)		(20%)		

Data are presented as absolute number (percentage) or as mean ± standard deviation (range)

*N*, number; *BMI*, body mass index; *EWL*, excess weight loss; *TWL*, total weight loss; *GERD*, gastroesophageal reflux disease

**Table 2:** Associated medical problems and their evolution after one anastomosis gastric bypass in obese patients with BMI >50 kg/m^2^

Comorbidity	% before OAGB	At 24-month follow-up	Rate of resolution
Arterial hypertension	30% (75/245)	Lost to follow-up = 9 No medications = 50 Treated = 16	76%
Diabetes	17% (41/245)	Lost to follow-up = 2 No medications = 36 Treated = 3	92%
OSAS	29% (70/245)	Lost to follow-up = 2 No medications = 66 Treated = 2	97%
Dyslipidemia	17% (41/245)	Lost to follow-up = 1 No medications = 39 Treated = 1	98%

*OSAS*, obstructive sleep apnea syndrome

In the column “%before OAGB”, the data are reported as % (number of patients having the comorbidity/total number of patients)

### Early Postoperative Complications

Postoperative mortality was not observed. Postoperative morbidity occurred in 14 (5.7%) patients (Table 3). Five of the patients had upper or lower GI bleeding without hemodynamic impairment and received red blood cell transfusions. Two patients had anastomotic bleeding following hypertensive crises, with hemodynamic impairment, and underwent upper GI endoscopy and treatment of the source of anastomotic bleeding (in one case with adrenaline injection, in the other one with diathermic loop). Three patients had pneumonia. Three patients had an anastomotic leak or perianastomotic abscess and were treated by antibiotics and fasting (two patients) or needed re-laparoscopic exploration (one patient) with Kher drain (T-tube) placement into the fistulous orifice and drainage, as described previously [20]. One patient had phlebitis.

**Table 3:** Postoperative morbi-mortality and late complications after one anastomosis gastric bypass (OAGB) for severe obesity with BMI >50 kg/m^2^

Early morbi-mortality	N (%)	Treatment
Postoperative death	0 (0%)	
Early postoperative complications	14 (5.7%)	
Gastro-intestinal bleeding	7 (2.9%)	5 = red blood cells transfusions
		2 = upper GI endoscopy and treatment of the bleeding source
Pneumonia	3 (1.2%)	Antibiotics
Anastomotic leak/perianastomotic abscess	3 (1.2%)	2 = antibiotics and fasting
		1 = re-laparoscopic exploration with Kher placement into the fistulous orifice and drainage,
Phlebitis	1 (0.4%)	Medical treatment
Late complications	N (%)	Treatment
Late postoperative complications	22 (9.0%)	
Internal hernia	6 (2.4%)	Surgery
Anastomotic ulcer	3 (1.2%)	1 = surgery
		2 = medical treatment
Additional surgery for insufficient	2 (0.8%)	1 = 2-step conversion into SADI
Weight loss		1 = calibration band added
Long-term deaths	2 (0.8%)	1 = colon cancer at 14 months
GERDrequiring conversion to RYGB	3 (1.2%)	1 = myocardial infarction at 18 months
Chronic diarrhea	4 (1.6%)	Medical treatment
Glycemic troubles	2 (0.8%)	Medical treatment
Overall complications*	36 (14.7%)	

*OAGB*, one anastomosis gastric bypass; *GERD*, gastroesophageal reflux disease; *SADI*, single anastomosis duodeno-ileal bypass; *RYGB*, Roux-en-Y gastric bypass; *GI*, gastrointestinal

*Early + late complications

### Long-Term Complications

Conversion to RYGB was needed in three (1.2%) patients for reflux that was resistant to medical treatment (Table 3). Six patients (2.4%) had reoperation for an internal hernia during follow-up. Anastomotic ulcers occurred in three (1.2%) patients. Only two patients (0.8%) underwent a second bariatric surgery for insufficient weight loss. One of the patients was treated by conversion to SADI in two steps (SG followed by duodeno-ileal anastomosis). The patient lost further weight but developed severe malnutrition and died from this complication. The other patient had gastric pouch banding with no complications. Total complications rate (early + late) was 14.7%.

### Long-Term Outcomes

Weight loss outcomes are reported in Table 1. At 24-month follow-up, weight loss outcomes were available for 184 patients, at 60 months for 79.

Twenty-three female patients became pregnant and delivered during follow-up. Compliance with vitamin treatment was observed in 146 (83%) patients at 24 months of follow-up (results available for 176 patients). Nutritional blood tests at 24 months are reported in Table 4. Intravenous iron injections were administered to three patients (1.2%).

**Table 4:** Blood test results before one anastomosis gastric bypass (OAGB) for severe obesity with BMI > 50 kg/m^2^ and at 24-month follow-up

Biochemical variables (reference values)	Before OAGB (n = 245)	% of abnormal results	At 24-month follow-up (n = 176)	% of abnormal results	p
Hemoglobin (12–16 g/L)	13.9 ± 1.25 (10.5–20.7) (n = 242)	4.9%	13.4 ± 1.42 (8.9–18,1) (n = 125)	9.6%	<0.0001
Albumin (35–52 g/L)	38.5 ± 3.9 (29–47) (n=187)	14.9%	39,3 ± 4 (26–48) (n = 112)	9.8%	0.534
Ferritin (15–150 μg/L)	175 ± 153.6 (9.4–979) (n = 198)	1.5%	10.8 ± 115.7 (5–860) (n = 119)	11.7%	0.001
Prealbumin (0.2–0.4 g/L)	0.25 ± 0.05 (0.15–0.45) (n = 163)	13.4%	0.23 ± 0.04 (0.14–0.44) (n = 103)	18.4%	0.0001
Vitamin A (1.72–2.52 μmol/L)	2.12 ± 0.5 (1.09–3.4) (n=91)	17%	1.74 ± 0.45 (0.8–3.39) (n = 98)	39.7%	0.0001
Vitamin B9 (10–79 ng/L)	14 ± 5.6 (4–42) (n = 190)	17.8%	36.5 ± 83.8 (4–814) (n=111)	10.8%	0.0145
Vitamin B12 (145–569 pmol/L )	329.3 ± 131.2 (91–942) (n = 197)	0.5%	270 ± 123 (72–876) (n = 117)	11.1%	0.053
Vitamin D (75–150 nmol/L )	36.2 ± 20 (3–107) (n = 193)	92.7%	69.1 ± 28.3 (11–192) (n = 118)	52.5%	< 0.0001
Calcium (2.15–2.5 mmol/L)	2.36 ± 0.13 (2.03–3.35) (n = 193)	3%	2.29 ± 0.12 (1.7–2.53) (n = 120)	8.3%	0.0032

The p are referred to the comparison between the results before OAGB (column “before OAGB”) and after 24 months (column “at 24-month follow-up”)

*n* number of patients

## Discussion

The treatment of severe obesity with BMI > 50 kg/m^2^ represents one of the main challenges of bariatric surgery. The choice of the optimal surgical procedure is debated and should take into account the technical issues related to higher BMIs, the rates of perioperative and postoperative morbidity, and the expected efficacy in terms of associated medical problems resolution and weight loss outcomes.

In the literature, two approaches have been proposed, consisting of stand-alone procedures (with the aim of proposing only one bariatric surgery) or two-step strategies including a bridging step followed by a second intervention [7, 27]. Two-step strategies were well described by a recent meta-analysis of 13 studies involving 550 patients and a mean BMI of 61.26 kg/m^2^ [7], undergoing a first intervention of laparoscopic SG, intragastric balloon, and liquid low calorie diet program. SG and diet were effective; in particular, SG guaranteed a BMI reduction of 15.2 kg/m^2^. However, long-term results are lacking in this meta-analysis and the literature.

Among bariatric surgeries performed as a stand-alone procedure, data have been published on SG, RYGB, OAGB, BPD-DS, and SADI in the setting of severe obesity with BMI > 50 kg/m^2^. The study by Nasser et al. [28] analyzed the data of 356, 621 patients who received SG or RYGB (65, 565 with BMI > 50, 18, 861 with BMI > 60). Patients with BMI > 50 kg/m^2^ had increased morbidity and mortality compared to those with morbid obesity. Wang and colleagues [29] compared SG and RYGB through a meta-analysis. In their study, RYGB was associated with a higher %EWL at 12 months compared with SG; however, no significant differences were found at 24 months. A systematic review by Parmar et al. [30], which included 318 patients, described the results of OAGB for severe obesity with BMI > 50 kg/m^2^, showing the safety efficacy of OAGB in this setting, with a leak rate of 0%, mortality of 0.31%, and 60 months %EWL of 90.75%. A single-institution series demonstrated the safety and efficacy of bariatric surgery to treat patients with BMI > 50 kg/m^2^ [31]. In some studies, SG and RYGB were comparable at 3-year follow-up [32], whereas others reported better results for RYGB at 1- and 2-year follow-up compared to SG [33], and OAGB at 1 year was more effective than SG [34]. Bhandari et al. reported their experience in India in a retrospective study with 3-year follow-up. They reported better outcomes after banded SG, OAGB, and banded RYGB compared to standard RYGB and SG [35]. Other authors reported promising results for banded RYGB and OAGB [11, 12].

Similar postoperative and weight loss results have been reported for BPD/DS and SADI in a retrospective multicenter series [36], whereas Skogar et al. showed that patients with severe obesity with BMI > 50 kg/m^2^ had a better weight reduction and metabolic control with BPD/DS, at the cost of higher incidence of adverse events, compared to patients undergoing RYGB [14].

The analysis of the literature shows that most authors advocate bypasses (RYGB or OAGB) or biliopancreatic diversion (BPD-DS or SADI) to treat severe obesity with BMI > 50 kg/m^2^ because they seem to be more effective than SG in this setting. Biliopancreatic diversion seems to be associated with a higher rate of postoperative morbidity than gastric bypass even if some authors advocate that it might guarantee higher weight loss in the setting of severe obesity with BMI > 50 kg/m^2^ than RYGB. Banding of the OAGB or RYGB may be useful in increasing the efficacy of these procedures. SG may be useful as a first step procedure when more complex procedures are too difficult because the BMI of the patient is high. However, it should be noted that the majority of previous studies is limited by short or incomplete follow-up, or by the small number of included patients.

The present study demonstrates that OAGB is feasible and associated with sustained weight loss, as a stand-alone bariatric procedure to treat people with severe obesity with BMI > 50 kg/m^2^. In experienced hands, the postoperative complication rate was as low as 5.7%, no mortality was observed, and weight loss outcomes were very satisfying. Five-year results were available for 79 patients from a total number of 245; %EWL was 78.1; and %TWL was 41.9. The effect on associated medical problems was remarkable, with the resolution rate ranging from 74% for arterial hypertension to 93% for obstructive sleep apnea syndrome. Diabetes was resolved in 88% of cases.

The present study is one of the most relevant single-center studies if we consider the number of patients and the follow-up data. We confirm that malnutrition requiring hospitalization or surgery was not observed using a 150-cm biliopancreatic loop, as we described previously [24]. However, we observed after a 24-month follow-up a slight increase in the rate of abnormal pre-albumin, from 13.4% before OAGB to 18.4% at 2-year follow-up.

Among long-term complications, bile reflux that was resistant to medical treatment requiring conversion to RYGB was observed in 1.2% of patients, anastomotic ulcers in 1.2%, and surgery for an internal hernia in 2.4% of cases. Only two patients underwent revisional surgery for insufficient weight loss, one of them with inauspicious outcomes. In this patient, OAGB was converted to SADI with a common limb of 250 cm but severe malnutrition occurred, and the patient ultimately died (the patient was treated by another team). The other patient had an adjustable banding placed on the gastric pouch. In our opinion, OAGB with a 150-cm limb works well as an effective malabsorbitive procedure [24]. So, we do not recommend limb elongation in case of insufficient weight loss, fearing the risk of nutritional complications. We believe that the placement of an adjustable gastric band on the pouch is a safer option (we underline that these data are only based on personal experience, giving the lack of data in the literature).

The total rate of complications (early + late) was 14.7%, which is considered acceptable considering that it includes long-term complication.

The present series, which only included OAGB as a primary procedure for severe obesity with BMI > 50 kg/m^2^, leads to several considerations. OAGB with a 150-cm biliopancreatic limb in our experience has several advantages in the treatment of these patients. First, it may be proposed as a stand-alone procedure (since only one conversion for insufficient weight loss was needed). This is important in the effort to reduce hospitalizations, complications, and costs, which are expected to be higher in two or three-step strategies. Furthermore, a stand-alone procedure avoids the loss of patients between the first and second step. After the first operation, some patients are lost to follow-up, are contraindicated for medical or psychiatric reasons, or have some improvement without wanting a second procedure receiving only limited benefit from the two-step strategy. Second, it is a feasible and relatively “simple” technique, requiring only one anastomosis, which is important in this setting because higher BMI is associated with more technical difficulties. The senior author (AL) standardized a technique of OAGB with a 150-cm limb and a mechanical gastrojejunal anastomosis [24]. Third, but probably the most important aspect, OAGB is associated with sustained weight loss in these patients, as our results demonstrate.

The disadvantages of OAGB are not frequent, and in our opinion, they are outbalanced by the benefits, but they exist and include the possibility of long-term complications requiring a second intervention, such as bile reflux that is resistant to medical treatment, anastomotic ulcers, and internal hernia [37]. Furthermore, the procedure is not always feasible, with 10 out of 255 patients not undergoing OAGB for technical reasons.

The peculiarity of the present series is the length of the biliopancreatic limb, which was standardized at 150 cm, even for severe obese patients with BMI > 50 kg/m^2^, and was effective in terms of weight loss and safe with regard to nutritional complications (we did not observe patients requiring surgery or hospitalization for malnutrition in this series). While other authors measure the limb and modify its length according to the BMI of the patient [38], we chose to adopt a standardized length without measuring the bowel. In our opinion, the resolution of severe obesity is related to a number of factors, and it is simplistic to correlate it only to the biliopancreatic limb length. On the other hand, lengthening the limb may increase the risk of malnutrition without an increase in efficacy.

The present study demonstrates the efficacy and utility of OAGB with a biliopancreatic limb of 150 cm to treat severe obese patients with BMI > 50 kg/m^2^. However, we highlight the need to conduct randomized trials to compare OAGB with other bariatric procedures. Furthermore, the role of the band placement over the bypass pouch needs to be defined in the future.

### Limits

The present study is limited by its single-institution design, which limits the number of included patients. The study was an observational trial without a matched control or randomization. Furthermore, the results of a center with a large experience of OAGB and management of its complications [20, 39, 40] may not be reproducible in low-volume or less experienced centers. Third, during the surgeries, the entire bowel length was not measured, as only the biliopancreatic limb was routinely measured. Until 2015, blood tests including nutritional assessment were performed in our center during the first 24 months. After 24 months, the patients were referred to their general practitioner/endocrinologist/nutritionist for nutritional monitoring, which explains the lack of nutritional assessment beyond 2 years in the results.

## Conclusion

OAGB with a biliopancreatic limb of 150 cm is feasible and associated with sustained weight loss in the treatment of severe obesity with BMI > 50 kg/m^2^. Shot-term morbidity is low, and weight loss outcomes and resolution of associated medical problems are promising. Further randomized studies are needed to compare OAGB with the other bariatric procedures in this setting.

## References

[1] Sjöström L, Narbro K, Sjöström CD, Karason K, Larsson B, Wedel H, Lystig T, Sullivan M, Bouchard C, Carlsson B, Bengtsson C, Dahlgren S, Gummesson A, Jacobson P, Karlsson J, Lindroos AK, Lönroth H, Näslund I, Olbers T, Stenlöf K, Torgerson J, Ågren G, Carlsson LMS (2007). Effects of bariatric surgery on mortality in Swedish obese subjects. N Engl J Med.

[2] Wang YC, McPherson K, Marsh T, Gortmaker SL, Brown M (2011). Health and economic burden of the projected obesity trends in the USA and the UK. Lancet..

[3] Kuzminov A, Palmer AJ, Wilkinson S, Khatsiev B, Venn AJ (2016). Re-operations after secondary bariatric surgery: a systematic review. Obes Surg.

[4] Switzer NJ, Karmali S, Gill RS, Sherman V (2016). Revisional bariatric surgery. Surg Clin North Am.

[5] de Raaff CAL, Coblijn UK, de Vries N, Heymans MW, van den Berg BTJ, van Tets WF, van Wagensveld BA (2016). Predictive factors for insufficient weight loss after bariatric surgery: does obstructive sleep apnea influence weight loss?. Obes Surg.

[6] Higa K, Ho T, Tercero F, Yunus T, Boone KB (2011). Laparoscopic Roux-en-Y gastric bypass: 10-year follow-up. Surg Obes Relat Dis.

[7] Lee Y, Dang JT, Switzer N, Malhan R, Birch DW, Karmali S (2019). Bridging interventions before bariatric surgery in patients with BMI ≥ 50 kg/m2: a systematic review and meta-analysis. Surg Endosc.

[8] Topart P, Becouarn G, Ritz P (2010). Should biliopancreatic diversion with duodenal switch be done as single-stage procedure in patients with BMI ≥50 kg/m2?. Surg Obes Relat Dis.

[9] Topart P, Becouarn G, Finel J-B (2020). Is transit bipartition a better alternative to biliopancreatic diversion with duodenal switch for superobesity? Comparison of the early results of both procedures. Surg Obes Relat Dis.

[10] Vitiello A, Berardi G, Velotti N, De Palma GD, Musella M. Should sleeve gastrectomy be considered only as a first step in super obese patients? 5-year results from a single center. Surgical Laparoscopy, Endoscopy & Percutaneous Techniques [Internet]. 2020 Sep 21 [cited 2020 Dec 15];Publish Ahead of Print. Available from: https://journals.lww.com/10.1097/SLE.0000000000000866 10.1097/SLE.0000000000000866 32956334

[11] Miller KA, Radauer M, Buchwald JN, McGlennon TW, Ardelt-Gattinger E (2020). 5-year results of banded one-anastomosis gastric bypass: a pilot study in super-obese patients. Obes Surg.

[12] Romeijn MM, Leclercq WKG, Luijten AAPM, Janssen L, van Dielen FMH. Banded Roux-en-Y gastric bypass in patients with super morbid obesity (BRandY-study): protocol of a cohort study with 10 year follow-up. BMC Surgery [Internet]. 2020 Dec [cited 2020 Dec 15];20(1). Available from: https://bmcsurg.biomedcentral.com/articles/10.1186/s12893-020-00784-x 10.1186/s12893-020-00784-x PMC7275500 32503510

[13] Hidalgo M, Vilallonga R (2020). Ruiz de Godejuela AG, Rodríguez-Luna MR, Balibrea JM, Roriz-Silva R, et al. Effectiveness of laparoscopic sleeve gastrectomy in super-obese and non–super-obese patients. Surg Laparosc Endosc Percut Techn.

[14] Skogar ML, Sundbom M (2017). Duodenal switch is superior to gastric bypass in patients with super obesity when evaluated with the bariatric analysis and reporting outcome system (BAROS). Obes Surg.

[15] Peterson K, Anderson J, Boundy E, Ferguson L, Erickson K (2017). Rapid evidence review of bariatric surgery in super obesity (BMI ≥ 50 kg/m2). J Gen Intern Med.

[16] De Luca M, Tie T, Ooi G, Higa K, Himpens J, Carbajo M-A (2018). Mini gastric bypass-one anastomosis gastric bypass (MGB-OAGB)-IFSO position statement. Obes Surg.

[17] Parmar CD, Mahawar KK (2018). One anastomosis (mini) gastric bypass is now an established bariatric procedure: a systematic review of 12,807 Patients. Obes Surg.

[18] Haddad A, Fobi M, Bashir A, Al Hadad M, ElFawal MH, Safadi B (2019). Outcomes of one anastomosis gastric bypass in the IFSO Middle East North Africa (MENA) region. Obes Surg.

[19] Musella M, Susa A, Manno E, De Luca M, Greco F, Raffaelli M (2017). Complications following the mini/one anastomosis gastric bypass (MGB/OAGB): a multi-institutional survey on 2678 patients with a mid-term (5 years) follow-up. Obes Surg.

[20] Liagre A, Queralto M, Juglard G, Anduze Y, Iannelli A, Martini F (2019). Multidisciplinary management of leaks after one-anastomosis gastric bypass in a single-center series of 2780 consecutive patients. Obes Surg.

[21] Robert M, Espalieu P, Pelascini E, Caiazzo R, Sterkers A, Khamphommala L, Poghosyan T, Chevallier JM, Malherbe V, Chouillard E, Reche F, Torcivia A, Maucort-Boulch D, Bin-Dorel S, Langlois-Jacques C, Delaunay D, Pattou F, Disse E (2019). Efficacy and safety of one anastomosis gastric bypass versus Roux-en-Y gastric bypass for obesity (YOMEGA): a multicentre, randomised, open-label, non-inferiority trial. Lancet..

[22] Neuberg M, Blanchet M-C, Gignoux B, Frering V. Long-term outcomes after one-anastomosis gastric bypass (OAGB) in morbidly obese patients. Obes Surg. 2019 10.1007/s11695-019-04287-4 31760607

[23] Boyle M, Mahawar K (2020). One anastomosis gastric bypass performed with a 150-cm biliopancreatic limb delivers weight loss outcomes similar to those with a 200-cm biliopancreatic limb at 18 -24 months. Obes Surg.

[24] Liagre A, Debs T, Kassir R, Ledit A, Juglard G, Chalret du Rieu M, et al. One anastomosis gastric bypass with a biliopancreatic limb of 150 cm: weight loss, nutritional outcomes, endoscopic results, and quality of life at 8-year follow-up. Obes Surg. 2020 10.1007/s11695-020-04775-y 32562132

[25] HAS, Haute Autorite de Sante. Obésité : prise en charge chirurgicale chez l’adulte [Internet]. [cited 2020 Feb 3]. Available from: https://www.has-sante.fr/jcms/c_765529/fr/obesite-prise-en-charge-chirurgicale-chez-l-adulte

[26] Clavien PA, Barkun J, de Oliveira ML, Vauthey JN, Dindo D, Schulick RD, de Santibañes E, Pekolj J, Slankamenac K, Bassi C, Graf R, Vonlanthen R, Padbury R, Cameron JL, Makuuchi M (2009). The Clavien-Dindo classification of surgical complications: five-year experience. Ann Surg.

[27] Ball W, Raza SS, Loy J, Riera M, Pattar J, Adjepong S, Rink J (2019). Effectiveness of intra-gastric balloon as a bridge to definitive surgery in the super obese. Obes Surg.

[28] Nasser H, Ivanics T, Leonard-Murali S, Shakaroun D, Genaw J (2019). Perioperative outcomes of laparoscopic Roux-en-Y gastric bypass and sleeve gastrectomy in super-obese and super-super-obese patients: a national database analysis. Surg Obes Relat Dis.

[29] Wang Y, Song Y, Chen J, Zhao R, Xia L, Cui Y, Rao ZY, Zhou Y, Wu XT (2019). Roux-en-Y Gastric bypass versus sleeve gastrectomy for super super obese and super obese: systematic review and meta-analysis of weight results, comorbidity resolution. Obes Surg.

[30] Parmar CD, Bryant C, Luque-de-Leon E, Peraglie C, Prasad A, Rheinwalt K, Musella M (2019). One anastomosis gastric bypass in morbidly obese patients with BMI ≥ 50 kg/m2: a Systematic review comparing it with Roux-En-Y gastric bypass and sleeve gastrectomy. Obes Surg.

[31] Andalib A, Alamri H, Almuhanna Y, Bouchard P, Demyttenaere S, Court O. Short-term outcomes of revisional surgery after sleeve gastrectomy: a comparative analysis of re-sleeve, Roux en-Y gastric bypass, duodenal switch (Roux en-Y and single-anastomosis). Surgical Endoscopy [Internet]. 2020 Aug 11 [cited 2020 Sep 22]; Available from: http://link.springer.com/10.1007/s00464-020-07891-z 10.1007/s00464-020-07891-z 32780238

[32] Hong J, Park S, Menzo EL, Rosenthal R (2018). Midterm outcomes of laparoscopic sleeve gastrectomy as a stand-alone procedure in super-obese patients. Surg Obes Relat Dis.

[33] Bettencourt-Silva R, Neves JS, Pedro J, Guerreiro V, Ferreira MJ, AMTCO Group (2019). Comparative effectiveness of different bariatric procedures in super morbid obesity. Obes Surg.

[34] Singla V, Aggarwal S, Singh B, Tharun G, Katiyar V, Bhambri A (2019). Outcomes in Super obese patients undergoing one anastomosis gastric bypass or laparoscopic sleeve gastrectomy. Obes Surg.

[35] Bhandari M, Ponce de Leon-Ballesteros G, Kosta S, Bhandari M, Humes T, Mathur W (2019). Surgery in patients with super obesity: medium-term follow-up outcomes at a high-volume center. Obesity..

[36] Pereira AM, Guimarães M, Pereira SS, Ferreira de Almeida R, Monteiro MP, Nora M (2021). Single and dual anastomosis duodenal switch for obesity treatment: a single-center experience. Surg Obes Relat Dis.

[37] Petrucciani N, Martini F, Kassir R, Juglard G, Hamid C, Boudrie H, et al. Internal hernia after one anastomosis gastric bypass (OAGB): lessons learned from a retrospective series of 3368 consecutive patients undergoing OAGB with a biliopancreatic limb of 150 cm. Obes Surg. 2021 10.1007/s11695-021-05269-1 PMC8113202 33830446

[38] Soong T-C, Almalki OM, Lee W-J, Ser K-H, Chen J-C, Wu C-C, Chen SC (2019). Measuring the small bowel length may decrease the incidence of malnutrition after laparoscopic one-anastomosis gastric bypass with tailored bypass limb. Surg Obes Relat Dis.

[39] Kassir R, Petrucciani N, Debs T, Juglard G, Martini F, Liagre A. Conversion of one anastomosis gastric bypass (OAGB) to Roux-en-Y gastric bypass (RYGB) for biliary reflux resistant to medical treatment: lessons learned from a retrospective series of 2780 consecutive patients undergoing OAGB. Obes Surg 2020;30(6):2093–8. 10.1007/s11695-020-04460-0 10.1007/s11695-020-04460-0 32052289

[40] Debs T, Petrucciani N, Kassir R, Juglard G, Gugenheim J, Iannelli A, Martini F, Liagre A (2020). Laparoscopic conversion of sleeve gastrectomy to one anastomosis gastric bypass for weight loss failure: mid-term results. Obes Surg.

